# Catalyst-free decarboxylation of 4-hydroxycinnamic acids: efficient synthesis of 4-vinylphenols

**DOI:** 10.1098/rsos.220014

**Published:** 2022-04-27

**Authors:** Qian Yang, Youjuan Li, Huanhuan Liu, Enhua Wang, Mei Peng, Tingfei Deng, Xiong Pan, Zhongsheng Luo, Yanfang Yan, Lishou Yang, Xiaosheng Yang

**Affiliations:** ^1^ State Key Laboratory of Functions and Applications of Medicinal Plants, Guizhou Medical University, Guiyang 550014, People's Republic of China; ^2^ The Key Laboratory of Chemistry for Natural Products of Guizhou Province and Chinese Academy of Sciences, Guiyang 550014, People's Republic of China; ^3^ Department of Medicine and Food, Guizhou Vocational College of Agriculture, Guiyang 550041, People's Republic of China

**Keywords:** catalyst-free, decarboxylation, 4-hydroxycinnamic acids, 4-vinylphenols

## Abstract

We report herein an efficient protocol for the synthesis of 4-vinylphenols by a catalyst-free decarboxylation of *trans*-4-hydroxycinnamic acids. A variety of 4-vinylphenols has been synthesized in moderate to excellent yields. This protocol also features no polymerization.

## 1. Introduction

**Figure 1.**
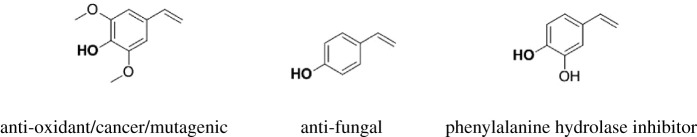
Selected bioactive 4-vinylphenols.

4-Vinylphenols are of both natural and biological interest. They are part of a large number of significant natural products such as 4-vinylphenol, 4-vinylcatechol, 2,6-dimethoxy-4-vinylphenol and many others [1–5]. Most of these compounds display varied bioactivities such as anti-oxidant [6–10], anti-mutagenic [9], anti-fungal [11] and anti-cancer [12–14] properties (figure 1). They are also building blocks in the synthesis of bioactive compounds [15–17]. In addition, they are widely used in industry [18]. Therefore, the synthesis of 4-vinylphenols has gained widespread attention.

**Scheme 1.**
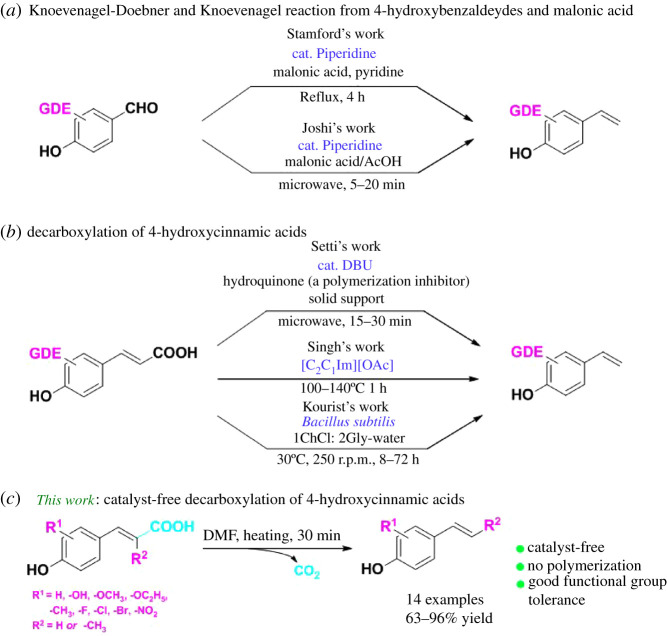
The most efficient approaches for the synthesis of 4-vinylphenols.

However, the susceptibility of the hydroxy function toward polymerization often results in the formation of polymers [19]. To overcome this barrier chemical [20–27] and biological [28–32] protocols have been developed, but most of them suffer from narrow substrate scope. To the best of our knowledge, the most efficient synthetic routes reported for the preparation of 4-vinylphenols involve (i) piperidine-catalysed Knoevenagel–Doebner [20] and Knoevenagel reaction [21] from 4-hydroxybenzaldeydes and malonic acid (scheme 1*a*); (ii) decarboxylation of 4-hydroxycinnamic acids using DBU [22], [C_2_C_1_Im][OAc] [23] or *Bacillus subtilis* [28] (scheme 1*b*). But these methods only test 4-hydroxycinnamic acids bearing electron-donating groups (EDG) [20–23,28] and suffer some disadvantages such as the use of a base catalyst (Stamford/Joshi/Setti/Singh's work), the addition of polymerization inhibitor (Setti's work), need of microwave-assisted (Joshi/Setti's work) and long reaction times (Stamford/Kourist's work). Several catalyst-free decarboxylation methods of cinnamic acids have been reported, however, they need using some sort of biocatalysts [29,33,34].

Herein, we report an efficient procedure for the preparation of 4-vinylphenols from *trans*-4-hydroxycinnamic acids by a catalyst-free decarboxylative reaction without any additive (scheme 1*c*). This approach tolerates 4-hydroxycinnamic acids bearing electron-donating/withdrawing groups and containing substituents on the double bond. Additionally, it can effectively inhibit the polymerization in the absence of inhibitor.

## 2. Results and discussion

Our initial studies were carried out with readily available 4-hydroxycinnamic acid **1a** as a test substrate. **1a** in DMF was stirred at 200°C for 60 min under an air atmosphere to give the desired product **2a** in 15% yield (table 1, entry 1). To further improve the reaction yield, control experiment was performed. To our delight, we found that decreasing the reaction time to 30 or 40 min had obvious effect on the reaction (table 1, entries 2–3). However, a decrease in the yield was observed upon further decreasing the reaction time (table 1, entry 4). Subsequently, we investigated the effect of solvents on the efficiency of the decarboxylation. Unfortunately, the reaction performed in DMA, ethylene glycol, DMSO, 1,4-dioxane or DCE exerted detrimental effect on the yield (table 1, entries 5–9). We tried to lower the temperature but failed. The reaction was conducted under microwave irradiation at 100°C, yielding **2a** only in 45% yield (table 1, entry 10). Therefore, the optimized reaction conditions were determined as **1a** (0.2 mmol) in DMF (1 ml) at 200°C for 30 min under an air atmosphere (table 1, entry 3). With the optimized reaction conditions in hand, a gram scale reaction was carried out and provided the product **2a** in 87% yield (table 1, entry 11).

**Table 1.** Optimization of the reaction conditions.^a^
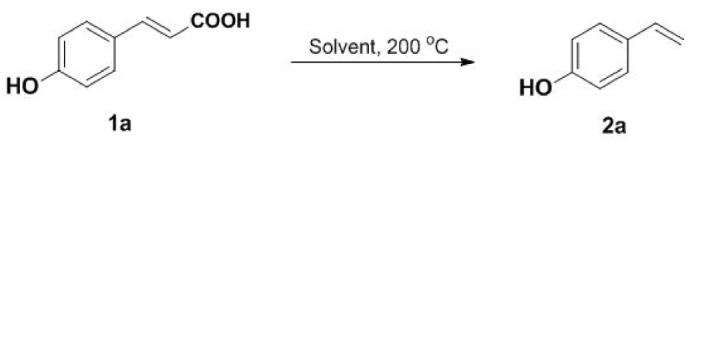

entry	time (min)	solvent	yield **2a** (%)^b^
1	60	DMF	15
2	40	DMF	85
3	30	DMF	89
4	20	DMF	77
5	30	DMA	23
6	30	ethylene glycol	61
7	30	DMSO	30
8	30	1,4-dioxane	/
9	30	DCE	/
10^c^	30	DMF	45
11^d^	30	DMF	87

^a^Reaction conditions: **1a** (0.2 mmol), solvent (1 ml).

^b^Isolated yield.

^c^Reaction conditions: **1a** (0.2 mmol), solvent (1 ml), under microwave irradiation at 100**°**C.

^d^Reaction conditions: **1a** (7 mmol), solvent (25 ml).

**Scheme 2.**
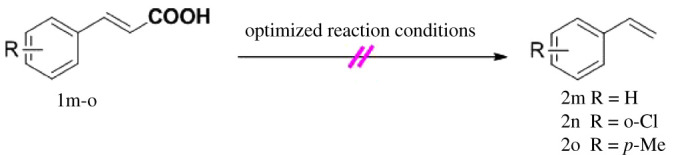
Decarboxylation of cinnamic acids without 4-hydroxyl substituent.

Under the optimized conditions, the scope of this decarboxylation was then examined by varying 4-hydroxycinnamic acids **1** (table 2). It is apparent from table 2 that 4-vinylphenols bearing electron-donating/withdrawing groups were prepared in excellent yields (86–96%) and no polymers were detected under the given conditions. Specifically, we investigated the decarboxylation of some natural products such as *p*-coumaric acid (**1a**), caffeic acid (**1b**), ferulic acid (**1c**) and sinapinic acid (**1d**), achieving corresponding 4-vinylphenols in excellent yields (**2a**–**2d**). Meanwhile, employing methyl- or ethoxy-substituted 4-hydroxycinnamic acids (**1e**–**1g**) also afforded the products in high yields (**2e**–**2g**). However, electron-withdrawing group (NO_2_, F, Cl or Br) gave lower yields (**2 h**–**2l**). Moreover, 4-hydroxycinnamic acids containing methyl on the double bond (**1p** and **1q**) were also compatible with the current conditions, providing the corresponding products **2p** and **2q** in moderate yields (68% and 63%). The products **2p** and **2q** exist in the *E* forms, based on their NMR spectra and in accordance with literature report [35]. Next, we tested several cinnamic acids without 4-hydroxyl substituent under the optimized reaction conditions. Unfortunately, no corresponding vinylbenzenes were detected (scheme 2).

**Table 2.** Evaluation of substrate scope.^a^
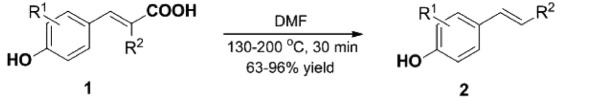

entry	substrates 1	temp. (^o^C)	products 2	yield (%)^b^
1	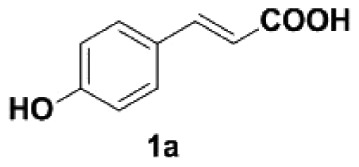	200	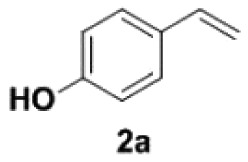	89
2	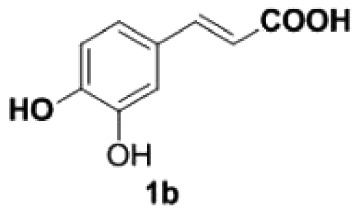	200	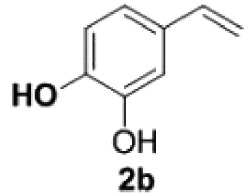	87
3	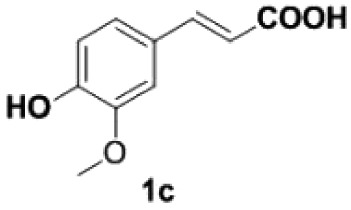	200	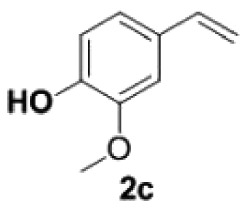	94
4	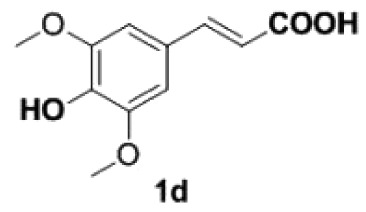	170	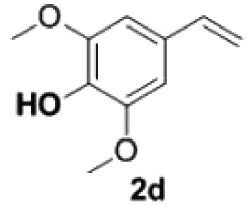	94
5	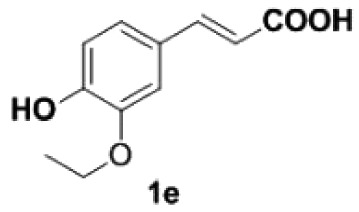	180	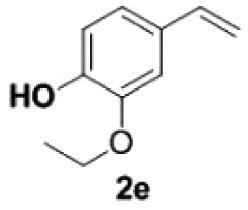	90
6	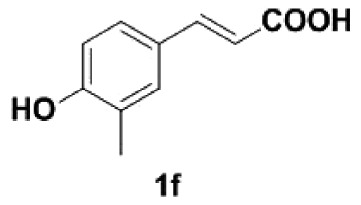	130	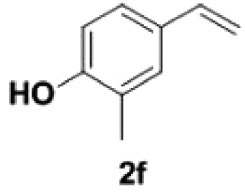	93
7	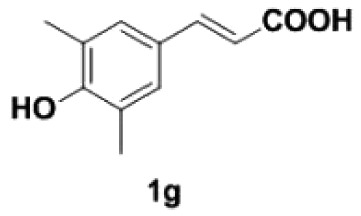	200	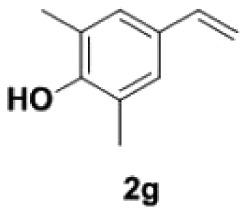	96
8	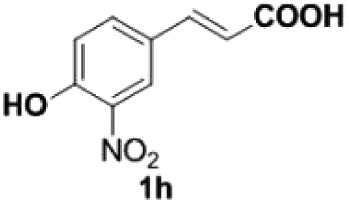	160	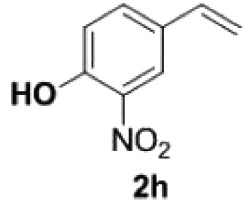	86
9	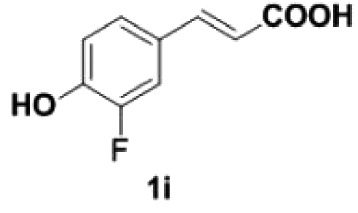	140	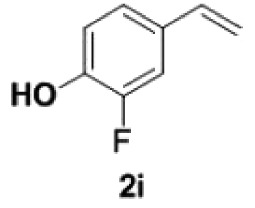	87
10	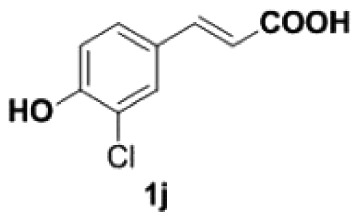	180	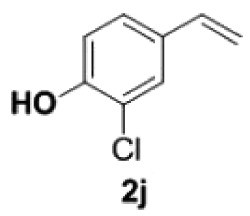	86
11	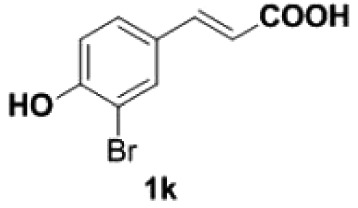	140	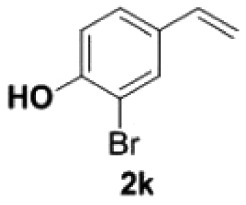	89
12	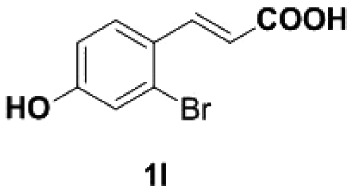	140	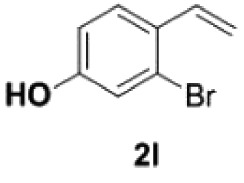	87
13	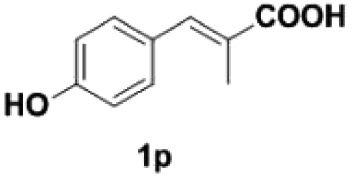	200	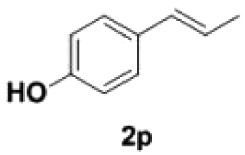	68
14	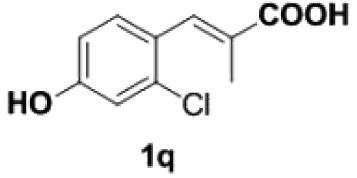	200	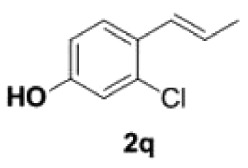	63

^a^Reaction conditions: **1** (0.2 mmol), DMF (1 ml).

^b^Isolated yield.

In addition, we found that the polymerization product **2aa** was isolated in 48% yield when the reaction time and temperature were increased (table 3, **2aa**). Subsequently, we tested another two substrates bearing electron-donating/withdrawing group (table 3, **1f** and **1l**). Moderate yields were obtained. Obviously, for electron-withdrawing group (Br) substituted substrate, related polymer was got in higher yield (table 3, 2ff versus 2ll).

**Table 3.** Synthesis of 4-vinylphenol dimers.^a^
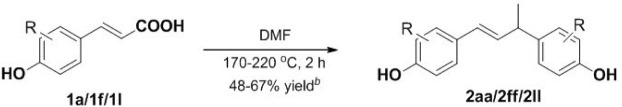

entry	substrates	temp. (^o^C)	dimers	yield (%)^b^
1	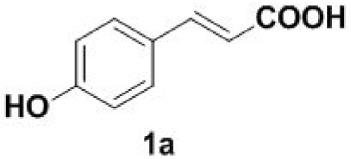	220	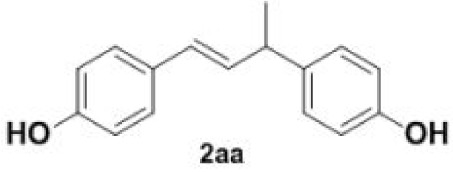	48
2	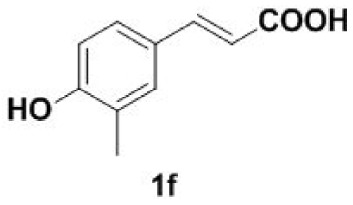	200	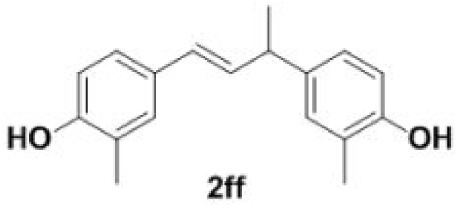	50
3	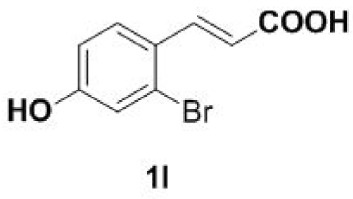	170	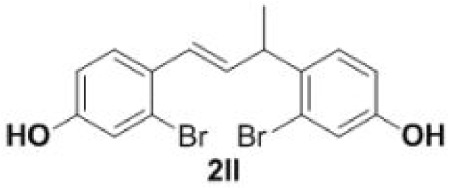	67

^a^Reaction conditions: **1a**/**1f**/**1 l** (0.2 mmol), DMF (1 ml).

^b^Isolated yield.

**Scheme 3.**
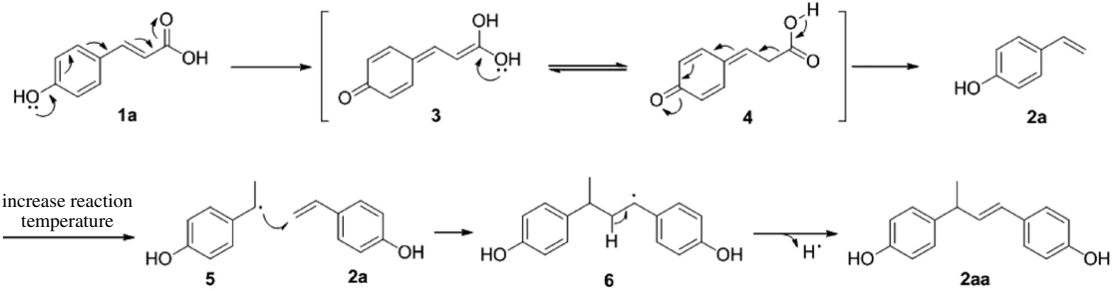
Plausible mechanism for the decarboxylation and polymerization.

Plausible mechanisms for the decarboxylation and polymerization of 4-hydroxycinnamic acids are depicted in scheme 3. Species **3** is formed under elevated reaction temperature. Finally, hydrogen transfer followed by release of a molecule of carbon dioxide ensures the formation of 4-vinylphenol **2a**. Radical polymerization of styrene initiated at higher reaction temperature, yielding intermediate **6** followed by expelling hydrogen radical leads to the polymerization product **2aa**.

## 3. Conclusion

In summary, we have developed an efficient method for the preparation of 4-vinylphenols via a catalyst-free decarboxylation of 4-hydroxycinnamic acids. This method features good functional group tolerance and no polymerization. However, corresponding polymers were obtained in moderate yields when under harsher reaction conditions.

## 4. Experimental section

### 4.1. General information

Unless otherwise noted, all reagents, catalysts and solvents were purchased from commercial suppliers and used without further purification. Column chromatography was performed with silica gel (200–300 mesh). Melting points were determined using a X-4 melting point apparatus with microscope. The IR spectra were recorded with Mattson FTIR spectrometer 5000. Absorption maxima were measured in cm^−1^. ^1^H and ^13^C NMR spectra were achieved on a Bruker Avance 600 MHz spectrometer (^1^H 600 MHz; ^13^C 151 MHz; ^19^F 565 MHz) in CDCl_3_, CD_3_OD, DMF-*d_7_*, DMSO-*d6*. High-resolution mass spectra were measured on a ThermoFish QE Focus facility. Thin-layer chromatographies were done on pre-coated silica gel 60F254 plates (Merck).

### 4.2. General procedure for the synthesis of 4-vinylphenols (**2a**–**2l** and **2p**–**2q**)

#### 4.2.1. Procedure for 4-vinylphenols bearing electron-donating groups (**2a–2g** and **2p**)

To a stirred solution of DMF (1 ml) was added 4-hydroxycinnamic acids (**1a**–**1g** and **1p**) (0.2 mmol) in 5 ml pressure-resistant reaction bottle. The reaction mixture was stirred at 130–200°C until the completion of the starting materials as monitored by TLC (30 min). The reaction mixture was quenched with water and extracted with ethyl acetate. The organic layer was dried over Na_2_SO_4_ and evaporated under reduced pressure. The resulting crude compound was purified by silica gel column chromatography to yield the pure product (**2a**–**2g** and **2p**), which was dissolved immediately in methanol for storage.

##### 4.2.1.1. 4-Vinylphenol **2a**

Light yellow solid, yield 89%, mp 72–74°C. IR (KBr plate): *ν*_max_ 3328, 3019, 2924, 2850, 1609, 1228, 834.^1^ H NMR (600 MHz, DMSO-*d6*) *δ* 7.29–7.23 (m, 2H), 6.72 (d, *J* = 8.5 Hz, 2H), 6.60 (dd, *J* = 17.6, 10.9 Hz, 1H), 5.57 (dd, *J* = 17.6, 0.9 Hz, 1H), 5.03 (dd, *J* = 10.9, 0.8 Hz, 1H). ^13^C NMR (151 MHz, DMSO-*d6*) *δ* 157.78, 136.86, 128.77, 127.89, 115.79, 111.12. HRMS-ESI (*m/z*): [M + H]^+^ calcd. for C_8_H_9_O: 121.06479; found, 121.06487.

##### 4.2.1.2. 2-Hydroxy-4-vinylphenol **2b**

Light yellow oil, yield 87%. IR (KBr plate): *ν*_max_ 3346, 2929, 1604, 1524, 1281, 1111, 815. ^1^H NMR (600 MHz, CD_3_OD) *δ* 6.94 (d, *J* = 1.8 Hz, 1H), 6.78 (dd, *J* = 8.1, 1.8 Hz, 1H), 6.75 (d, *J* = 8.1 Hz, 1H), 6.61 (dd, *J* = 17.6, 10.9 Hz, 1H), 5.56 (dd, *J* = 17.6, 0.9 Hz, 1H), 5.06 (dd, *J* = 10.9, 0.8 Hz, 1H). ^13^C NMR (151 MHz, CD_3_OD) *δ* 145.16, 144.98, 136.75, 130.03, 118.31, 114.82, 112.21, 109.34. HRMS-ESI (*m/z*): [M + H]^+^ calcd. for C_8_H_9_O_2_: 137.05971; found, 137.05946.

##### 4.2.1.3. 2-Methoxy-4-vinylphenol **2c**

Colourless oil, yield 94%. IR (KBr plate): *ν*_max_ 3411, 2924, 2852, 1603, 15 141 463, 1269, 817. ^1^H NMR (600 MHz, CDCl_3_) *δ* 6.95–6.91 (m, 2H), 6.87 (d, *J* = 8.1 Hz, 1H), 6.64 (dd, *J* = 17.5, 10.8 Hz, 1H), 5.65 (s, 1H), 5.59 (d, *J* = 17.5 Hz, 1H), 5.13 (d, *J* = 10.9 Hz, 1H), 3.91 (s, 3H). ^13^C NMR (151 MHz, CDCl_3_) *δ* 146.59, 145.64, 136.63, 130.28, 120.08, 114.35, 111.47, 108.01, 55.89. HRMS-ESI (*m/z*): [M + H]^+^ calcd. for C_9_H_11_O_2_: 151.07536; found, 151.07515.

##### 4.2.1.4. 2,6-Dimethoxy-4-vinylphenol **2d**

Yellow oil, yield 94%. IR (KBr plate): *ν*_max_ 3144, 2938, 2844, 1605, 1462, 1213, 1115, 837. ^1^H NMR (600 MHz, CDCl_3_) *δ* 6.65 (s, 2H), 6.61 (dd, *J* = 17.5, 10.9 Hz, 1H), 5.60 (d, *J* = 17.5 Hz, 1H), 5.56 (s, 1H), 5.15 (d, *J* = 10.8 Hz, 1H), 3.90 (s, 6H). ^13^C NMR (151 MHz, CDCl_3_) *δ* 147.06, 136.83, 134.76, 129.18, 111.87, 102.9, 56.26. HRMS-ESI (*m/z*): [M + H]^+^ calcd. for C_10_H_13_O_3_: 181.08592; found, 181.08562.

##### 4.2.1.5. 2-Ethoxy-4-vinylphenol **2e**

White solid, yield 90%, mp 125–127°C. IR (KBr plate): *ν*_max_ 3436, 2979, 2929, 1606, 1513, 1237, 1122, 823. ^1^H NMR (600 MHz, CD_3_OD) *δ* 6.99 (d, *J* = 1.7 Hz, 1H), 6.85 (dd, *J* = 8.1, 1.7 Hz, 1H), 6.74 (d, *J* = 8.1 Hz, 1H), 6.61 (dd, *J* = 17.6, 10.9 Hz, 1H), 5.56 (d, *J* = 17.6 Hz, 1H), 5.04 (d, *J* = 10.9 Hz, 1H), 4.10 (q, *J* = 7.0 Hz, 2H), 1.42 (t, *J* = 7.0 Hz, 3H). ^13^C NMR (151 MHz, CD_3_OD) *δ* 146.77, 146.59, 136.73, 129.95, 119.41, 114.84, 110.25, 109.67, 64.19, 13.76. HRMS-ESI (*m/z*): [M + H]^+^ calcd. for C_10_H_13_O_2_: 165.09101; found, 165.09073.

##### 4.2.1.6. 2-Methyl-4-vinylphenol **2f**

Yellow oil, yield 93%. IR (KBr plate): *ν*_max_ 3375, 2922, 2850, 1599, 1461, 1267, 822. ^1^H NMR (600 MHz, DMF-*d_7_*) *δ* 7.24 (d, *J* = 1.5 Hz, 1H), 7.14 (dd, *J* = 8.2, 2.1 Hz, 1H), 6.87 (d, *J* = 8.2 Hz, 1H), 6.63 (dd, *J* = 17.6, 10.9 Hz, 1H), 5.60 (dd, *J* = 17.6, 1.0 Hz, 1H), 5.01 (dd, *J* = 10.9, 1.0 Hz, 1H), 2.18 (s, 3H). ^13^C NMR (151 MHz, DMF-*d_7_*) *δ* 158.04, 141.10, 133.70, 133.70, 130.49, 129.88, 121.53, 116.99, 34.39. HRMS-ESI (*m/z*): [M-H]^−^ calcd. for C_9_H_9_O: 133.06479; found, 133.06454.

##### 4.2.1.7. 2,6-Dimethyl-4-vinylphenol **2g**

Yellow oil, yield 96%. IR (KBr plate): *ν*_max_ 3436, 2924, 2853, 1600, 1202, 1148, 871. ^1^H NMR (600 MHz, CDCl_3_) *δ* 7.05 (s, 2H), 6.59 (dd, *J* = 17.6, 10.9 Hz, 1H), 5.58 (d, *J* = 17.6 Hz, 1H), 5.08 (d, *J* = 10.9 Hz, 1H), 4.65 (s, 1H), 2.25 (s, 6H). ^13^C NMR (151 MHz, CDCl_3_) *δ* 152.09, 136.46, 129.90, 126.59, 122.97, 111.16, 15.91. HRMS-ESI (*m/z*): [M + H]^+^ calcd. for C_10_H_13_O: 149.09609; found, 149.09592.

##### 4.2.1.8. (E)-1-(4-hydroxyphenyl)propene **2p**

Colourless oil, yield 68%. IR (KBr plate): *ν*_max_ 3388, 2964, 2927, 1615, 1558, 1507, 1457, 1239, 853.03, 688. ^1^H NMR (600 MHz, CDCl_3_) *δ* 7.24–7.20 (m, 2H), 6.80–6.77 (m, 2H), 6.35 (dd, *J* = 15.7, 1.6 Hz, 1H), 6.09 (dq, *J* = 15.7, 6.6 Hz, 1H), 1.87 (dd, *J* = 6.6, 1.7 Hz, 3H). ^13^C NMR (151 MHz, CDCl_3_) *δ* 154.77, 130.75, 130.32, 127.04, 123.36, 115.39, 18.39. HRMS-ESI (*m/z*): [M-H]^−^ calcd. for C_9_H_9_O: 133.06479; found, 133.06425.

#### 4.2.2. Procedure for 4-vinylphenols bearing electron-withdrawing groups (**2h–2l** and **2q**)

To a stirred solution of DMF (1 ml) was added 4-hydroxycinnamic acids (**1h**–**1l** and **1q**) (0.2 mmol) in 5 ml pressure-resistant reaction bottle. The reaction mixture was stirred at 140–200°C until the completion of the starting materials as monitored by TLC (30 min). The reaction mixture was quenched with water and extracted with dichloromethane. The dichloromethane layer was washed with pure water 2–3 times. The combined extract was dried over Na_2_SO_4_. The filtrate was evaporated under reduced pressure to yield the pure product (**2h**–**2l** and **2q**), which was dissolved immediately in methanol for storage.

##### 4.2.2.1. 2-Nitro-4-vinylphenol **2h**

Yellow oil, yield 86%. IR (KBr plate): *ν*_max_ 3418, 2925, 2853, 1627, 1536, 1322, 1260, 802. ^1^H NMR (600 MHz, CDCl_3_) *δ* 10.57 (s, 1H), 8.09 (d, *J* = 2.0 Hz, 1H), 7.67 (dd, *J* = 8.7, 2.1 Hz, 1H), 7.13 (d, *J* = 8.7 Hz, 1H), 6.65 (dd, *J* = 17.6, 10.9 Hz, 1H), 5.73 (d, *J* = 17.5 Hz, 1H), 5.32 (d, *J* = 10.9 Hz, 1H). ^13^C NMR (151 MHz, CDCl_3_) *δ* 154.59, 134.85, 134.11, 130.53, 122.42, 120.16, 114.96. HRMS-ESI (*m/z*): [M + H]^+^ calcd. for C_8_H_8_O_3_N: 166.04987; found, 166.04968.

##### 4.2.2.2. 2-Fluoro-4-vinylphenol **2i**

Colourless oil, yield 87%. IR (KBr plate): *ν*_max_ 3436, 2924, 2852, 1612, 1094. ^1^H NMR (600 MHz, CDCl_3_) *δ* 7.15 (dd, *J* = 11.8, 2.0 Hz, 1H), 7.06 (d, *J* = 8.3 Hz, 1H), 6.96–6.93 (m, 1H), 6.59 (dd, *J* = 17.5, 10.9 Hz, 1H), 5.61–5.58 (m, 1H), 5.56 (s, 1H), 5.17 (d, *J* = 10.8 Hz, 1H). ^13^C NMR (151 MHz, CDCl_3_) *δ* 151.15(237.72), 143.30(14.33), 135.46(2.19), 131.14(6.21), 123.03(2.74), 117.18, 112.80(18.79). HRMS-ESI (*m/z*): [M-H]^−^ calcd. for C_8_H_6_OF: 137.03972; found, 137.03989.

##### 4.2.2.3. 2-Chloro-4-vinylphenol **2j**

Yellow oil, yield 86%. IR (KBr plate): *ν*_max_ 3498, 2923, 2850, 1619, 1261, 1099, 804. ^1^H NMR (600 MHz, CDCl_3_) *δ* 7.38 (d, *J* = 2.0 Hz, 1H), 7.23 (dd, *J* = 8.4, 2.0 Hz, 1H), 6.98–6.96 (m, 1H), 6.59 (dd, *J* = 17.5, 10.9 Hz, 1H), 5.61 (d, *J* = 17.5 Hz, 1H), 5.58 (s, 1H), 5.18 (d, *J* = 10.9 Hz, 1H). ^13^C NMR (151 MHz, CDCl_3_) *δ* 150.92, 135.10, 131.60, 126.61, 126.36, 120.08, 116.21, 113.03. HRMS-ESI (*m/z*): [M-H]^−^ calcd. for C_8_H_6_OCl: 153.01017; found, 153.01038.

##### 4.2.2.4. 2-Bromo-4-vinylphenol **2k**

Colourless oil, yield 89%. IR (KBr plate): *ν*_max_ 3425, 2919, 2850, 1602, 1126, 618. ^1^H NMR (600 MHz, CDCl_3_) *δ* 7.52 (s, 1H), 7.28 (s, 1H), 6.97 (d, *J* = 8.4 Hz, 1H), 6.58 (dd, *J* = 17.5, 10.9 Hz, 1H), 5.61 (d, *J* = 17.5 Hz, 1H), 5.53 (s, 1H), 5.17 (d, *J* = 10.9 Hz, 1H). ^13^C NMR (151 MHz, CDCl_3_) *δ* 151.85, 134.94, 132.01, 129.65, 127.08, 116.03, 113.06, 110.44. HRMS-ESI (*m/z*): [M + H]^+^ calcd. for C_8_H_8_OBr: 198.97530; found, 198.97460.

##### 4.2.2.5. 3-Bromo-4-vinylphenol **2l**

Colourless oil, yield 87%. IR (KBr plate): *ν*_max_ 3405, 2925, 2850, 1605, 1228, 873, 596. ^1^H NMR (600 MHz, CDCl_3_) *δ* 7.44 (d, *J* = 8.5 Hz, 1H), 7.06 (d, *J* = 2.5 Hz, 1H), 6.97 (dd, *J* = 17.4, 10.9 Hz, 1H), 6.78 (dd, *J* = 8.5, 2.5 Hz, 1H), 5.57 (d, *J* = 17.4 Hz, 1H), 5.30 (s, 1H), 5.24 (d, *J* = 10.9 Hz, 1H). ^13^C NMR (151 MHz, CDCl_3_) *δ* 155.74, 135.03, 130.23, 127.48, 123.87, 119.45, 115.08, 114.62. HRMS-ESI (*m/z*): [M + H]^+^ calcd. for C_8_H_8_OBr: 198.97530; found, 198.97508.

##### 4.2.2.6. (E)-1-(2-chloro-4-hydroxyphenyl)propene **2q**

Colourless oil, yield 63%. IR (KBr plate): *ν*_max_ 3390, 2962, 2931, 2848, 1605, 1493, 1435, 1252, 1222, 1041, 963, 903, 854, 824, 691. ^1^H NMR (600 MHz, CDCl_3_) *δ* 7.38 (d, *J* = 8.5 Hz, 1H), 6.87 (d, *J* = 2.6 Hz, 1H), 6.73–6.68 (m, 2H), 6.11 (dq, *J* = 15.7, 6.6 Hz, 1H), 1.92 (dd, *J* = 6.6, 1.8 Hz, 3H). ^13^C NMR (151 MHz, CDCl_3_) *δ* 154.89, 132.80, 128.83, 127.44, 126.66, 126.60, 116.23, 114.48, 18.70. HRMS-ESI (*m/z*): [M-H]^−^ calcd. for C_9_H_8_OCl: 106.02582; found, 168.02541.

### 4.3. General procedure for the synthesis of 4-vinylphenol dimers (**2aa**/**2ff**/**2ll**)

To a stirred solution of DMF (1 ml) were added 4-hydroxycinnamic acids **1a**/**1f**/**1l** (0.2 mmol) in 5 ml pressure-resistant reaction bottle. The reaction mixture was stirred at 170–220°C until the completion of the starting materials as monitored by TLC (2 h). The reaction mixture was quenched with water and extracted with ethyl acetate. The organic layer was washed with saturated sodium bicarbonate solution, dried over Na_2_SO_4_ and evaporated under reduced pressure. The resulting crude compound was purified by silica gel column chromatography and dried by vacuum freeze-drying, affording the pure dimer (**2aa**/**2ff**/**2ll**). The pure product was dissolved immediately in methanol for storage.

#### 4.3.1. Dimer **2aa**

Light yellow oil, yield 48%. IR (KBr plate): *ν*_max_ 3328, 3021, 2962, 1610, 15 121 233,1171, 834. ^1^H NMR (600 MHz, CDCl_3_) *δ* 7.23 (d, *J* = 8.6 Hz, 2H), 7.13 (d, *J* = 8.5 Hz, 2H), 6.81–6.74 (m, 4H), 6.31 (d, *J* = 15.9 Hz, 1H), 6.20 (dd, *J* = 15.9, 6.8 Hz, 1H), 4.82 (s, 1H), 4.74 (s, 1H), 3.55 (p, *J* = 6.7 Hz, 1H), 1.41 (d, *J* = 7.0 Hz, 3H). ^13^C NMR (151 MHz, CDCl_3_) *δ* 154.69, 153.82, 138.13, 133.54, 130.63, 128.42, 127.56, 127.43, 115.37, 115.23, 41.64, 21.40. HRMS-ESI (*m/z*): [M + H]^+^ calcd. for C_16_H_17_O_2_: 241.12231; found, 241.12279.

#### 4.3.2. Dimer **2ff**

Light yellow oil, yield 50%. IR (KBr plate): *ν*_max_ 3416, 2960, 2921, 1611, 15 021 263,1114, 814. ^1^H NMR (600 MHz, CD_3_OD) *δ* 7.09 (s, 1H), 7.01 (dd, *J* = 8.2, 2.1 Hz, 1H), 6.97 (s, 1H), 6.90 (dd, *J* = 8.2, 2.1 Hz, 1H), 6.69 (dd, *J* = 15.1, 8.2 Hz, 2H), 6.25 (d, *J* = 15.9 Hz, 1H), 6.16 (dd, *J* = 15.8, 6.8 Hz, 1H), 4.62 (s, 2H), 3.46 (p, *J* = 7.2 Hz, 1H), 2.19 (s, 3H), 2.18 (s, 3H), 1.39 (d, *J* = 7.0 Hz, 3H). ^13^C NMR (151 MHz, CD_3_OD) *δ* 142.81, 141.78, 127.48, 123.49, 120.83, 120.65, 119.76, 119.27, 116.87, 116.30, 116.17, 116.06, 107.50, 107.46, 43.85, 25.47, 20.29, 20.21. HRMS-ESI (*m/z*): [M-H]^−^ calcd. for C_18_H_19_O_2_: 267.13796; found, 267.13870.

#### 4.3.3. Dimer **2ll**

Light yellow oil, yield 67%. IR (KBr plate): *ν*_max_ 3405, 2965, 2923, 1603, 1485, 1228, 1209, 875. ^1^H NMR (600 MHz, CD_3_OD) *δ* 7.40 (d, *J* = 8.6 Hz, 1H), 7.16 (d, *J* = 8.5 Hz, 1H), 7.04 (d, *J* = 2.5 Hz, 1H), 7.00 (d, *J* = 2.5 Hz, 1H), 6.80 (dd, *J* = 8.5, 2.5 Hz, 1H), 6.75 (dd, *J* = 8.6, 2.4 Hz, 1H), 6.69–6.64 (m, 1H), 6.17 (dd, *J* = 15.8, 6.3 Hz, 1H), 4.62 (s, 2H), 4.10–4.00 (m, 2H), 1.41 (d, *J* = 7.0 Hz, 3H). ^13^C NMR (151 MHz, CD_3_OD) *δ* 157.24, 156.27, 134.86, 134.08, 128.48, 128.34, 127.15, 127.09, 123.58, 123.02, 118.98, 118.68, 114.85, 114.80, 40.22, 19.49. HRMS-ESI (*m/z*): [M-H]^−^ calcd. for C_16_H_13_Br_2_O_2_: 394.92768; found, 394.92844.

## Data Availability

The datasets supporting this article have been uploaded as part of the electronic supplementary material [36].
